# Supplementary material for: Molecular interplay between ComEC domains allows for selective degradation of the non-translocating strand during natural transformation
Source: Nucleic Acids Res. 2025 Sep 23;53(18):gkaf932. doi: 10.1093/nar/gkaf932 (PMC12455585; doi:10.1093/nar/gkaf932)
Supplement: gkaf932_Supplemental_File [file gkaf932_supplemental_file.pdf]

Supplementary Information

Molecular interplay between ComEC domains allows for selective degradation of the non-translocating strand during natural transformation

Matthew J.M. Stedman[1,2,$], Sophie Deselaers[1,3,$], Sebastian A.G. Braus[1,4,$], Dianhong Wang[1,5,$], Maria Gregori Balaguer[1], Alvar D. Gossert[1,6], Manuela K. Hospenthal[1,7,*]

[1] Institute of Molecular Biology and Biophysics, ETH Zürich, Otto-Stern-Weg 5, 8093 Zürich, Switzerland

[$] These authors contributed equally

* Correspondence: manuela.hospenthal@mol.biol.ethz.ch

[2] ORCID: 0009-0009-7278-5349
[3] ORCID: 0009-0000-4290-7792
[4] ORCID: 0000-0002-1263-2107
[5] ORCID: 0000-0002-4337-4593
[6] ORCID: 0000-0001-7732-495X
[7] ORCID: 0000-0003-1175-6826

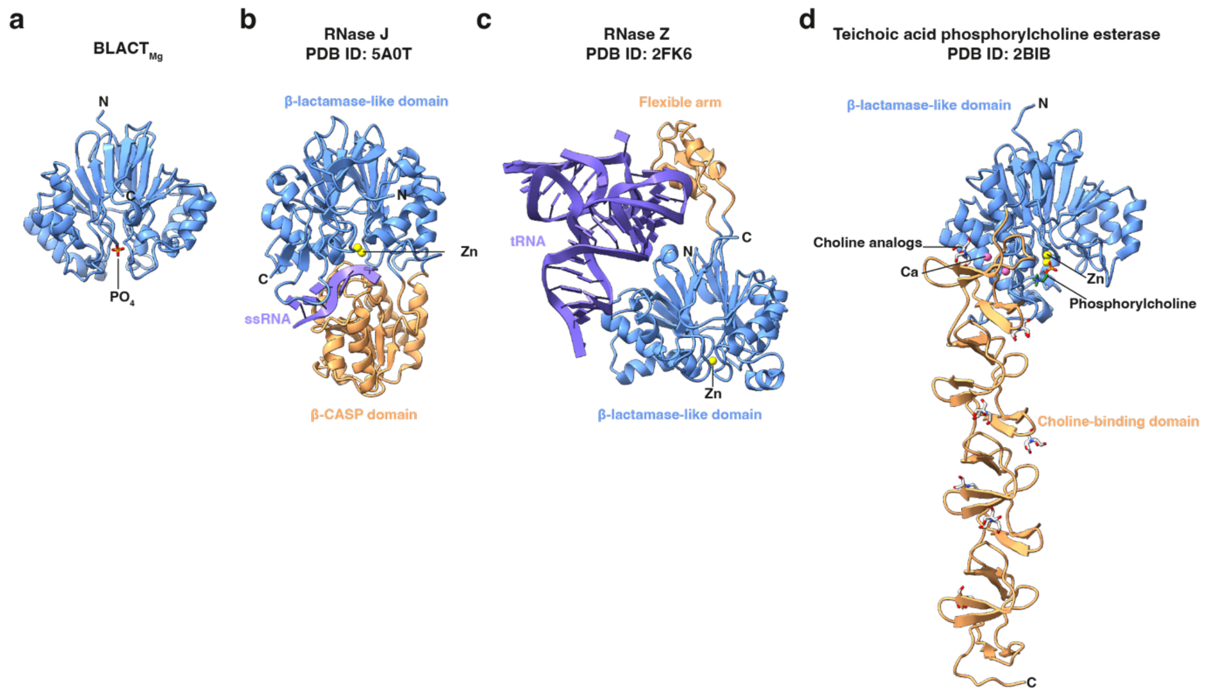

Fig. S1: Structural comparison of $BLACT_{Mg}$ homologues
a-d Structures of the β-lactamase-like domain from *Moorella glycerini* ($BLACT_{Mg}$) (this study) (**a**), RNase J from *Streptomyces coelicolor* (PDB ID: 5A0T) (1) (**b**), the RNase Z/tRNA complex from *Bacillus subtilis* (PDB ID: 2FK6) (2) (**c**), and teichoic acid phosphorylcholine esterase (Pce) from *Streptococcus pneumoniae* (PDB ID: 2BIB) (3) (**d**). Structures are shown in ribbon style and the β-lactamase-like domains, substrate binding domains, and RNA molecules are coloured blue, orange and purple, respectively. The Zn and Ca ions are represented by yellow and pink spheres, respectively. All ligands are labelled and shown in stick style.

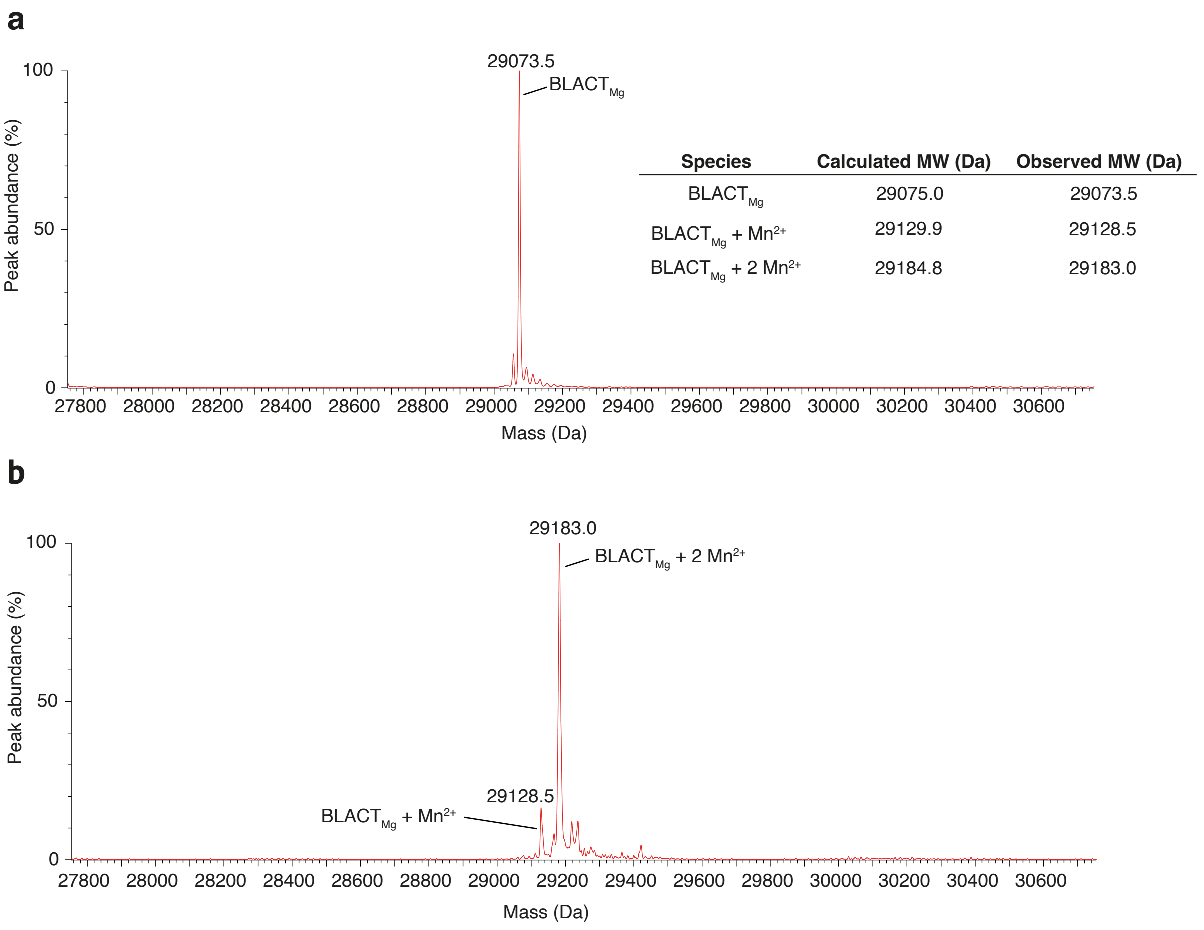

Species	Calculated MW (Da)	Observed MW (Da)
$BLACT_{Mg}$	29075.0	29073.5
$BLACT_{Mg}$ + Mn^{2+}	29129.9	29128.5
$BLACT_{Mg}$ + 2 Mn^{2+}	29184.8	29183.0

Fig. S2: The active site of $BLACT_{Mg}$ can coordinate two manganese ions
a, b Intact mass measurements of $BLACT_{Mg}$ without supplemented ions (**a**) and in the presence of 5 mM $MnCl_2$ (**b**). A table of theoretical molecular weights (MW) is shown as an inset. In the absence of supplemented $MnCl_2$, a peak corresponding to the expected mass of $BLACT_{Mg}$ alone is observed. Two peaks are observed in the presence of $MnCl_2$ corresponding to expected masses of $BLACT_{Mg}$ + Mn^{2+} and $BLACT_{Mg}$ + 2 Mn^{2+}. The peak corresponding to the fully coordinated state (+ 2 Mn^{2+}) is the most abundant peak.

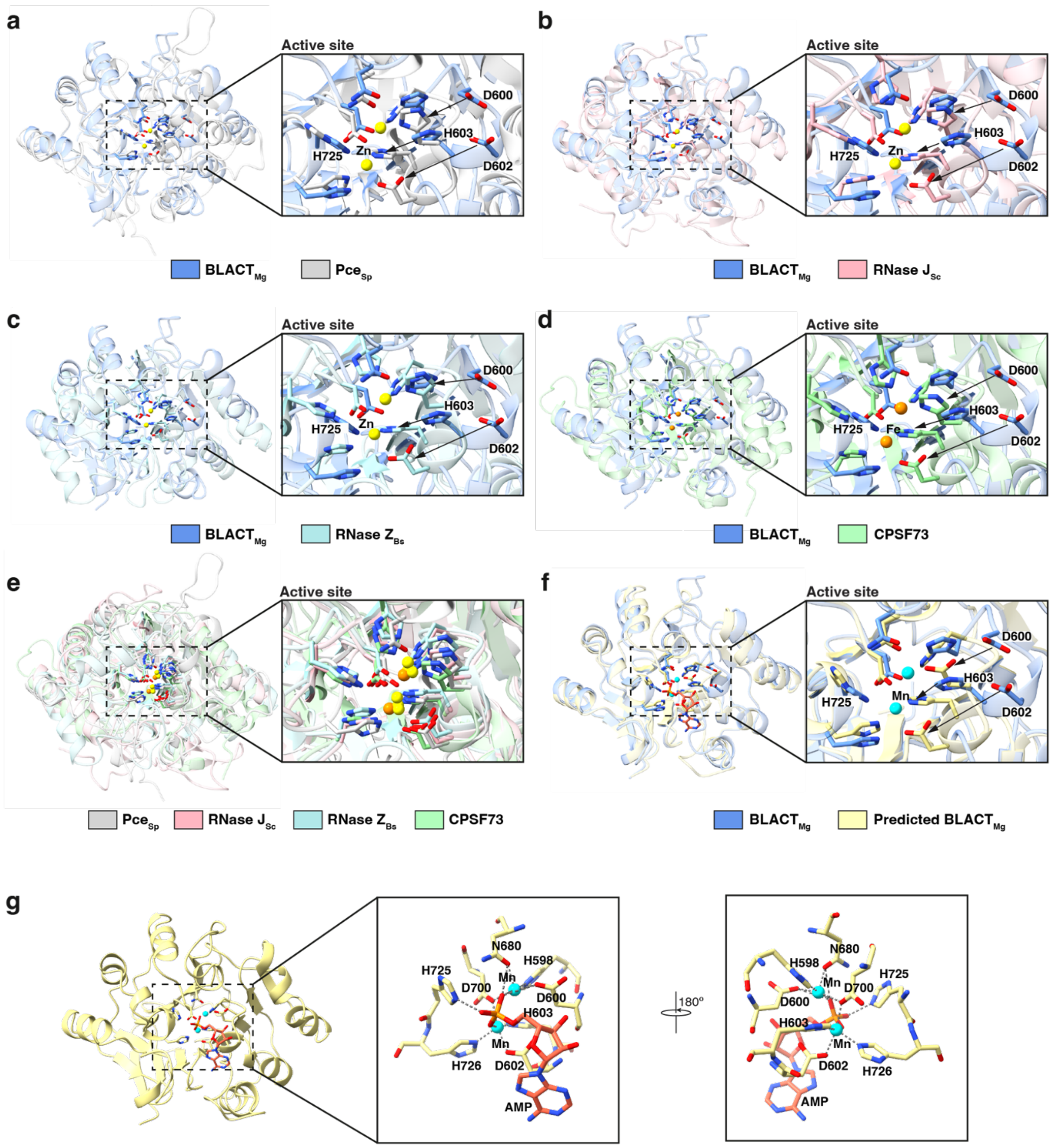

Fig. S3: The predicted catalytically competent active site of BLACT$_{Mg}$ in the presence of metal ions based on structural comparisons with homologues and modelling

a-d Superposition of BLACT$_{Mg}$ (blue, this study) and its homologues, including teichoic acid phosphorylcholine esterase (Pce) from *S. pneumoniae* (grey, PDB ID: 2BIB) (3) (**a**), RNase J from *S. coelicolor* (pink, PDB ID: 5A0T) (1) (**b**), RNase Z from *B. subtilis*, (cyan, PDB ID:1Y44) (4) (**c**), and human CPSF73 (green, PDB ID: 8T1R) (5) (**d**). Structures are displayed in ribbon representation and side chains of key residues are shown in stick representation with carbons coloured orange, oxygens red, and nitrogens blue. Zn, Fe and Mn ions are represented by yellow, orange and cyan spheres, respectively. The active site is indicated by a dashed box (left) and the zoomed-in is shown on the right. Rearrangements or key residues are indicated by arrows. **e** Superposition of BLACT$_{Mg}$ homologues. **f** Superposition of BLACT$_{Mg}$ (blue, this study) and the AlphaFold 3 (6) prediction of BLACT$_{Mg}$ coordinated by two Mn ions (cyan) and bound to adenosine monophosphate (AMP) (orange). In the zoomed-in view of the active site,

key residue rearrangements are shown, while AMP has been removed for clarity. **g** Interaction network within the active site of the predicted $BLACT_{Mg}$ structure (yellow) coordinated by two Mn ions (cyan) and AMP (orange). Key residues and AMP are shown as sticks and hydrogen bonds are indicated by dashed lines.

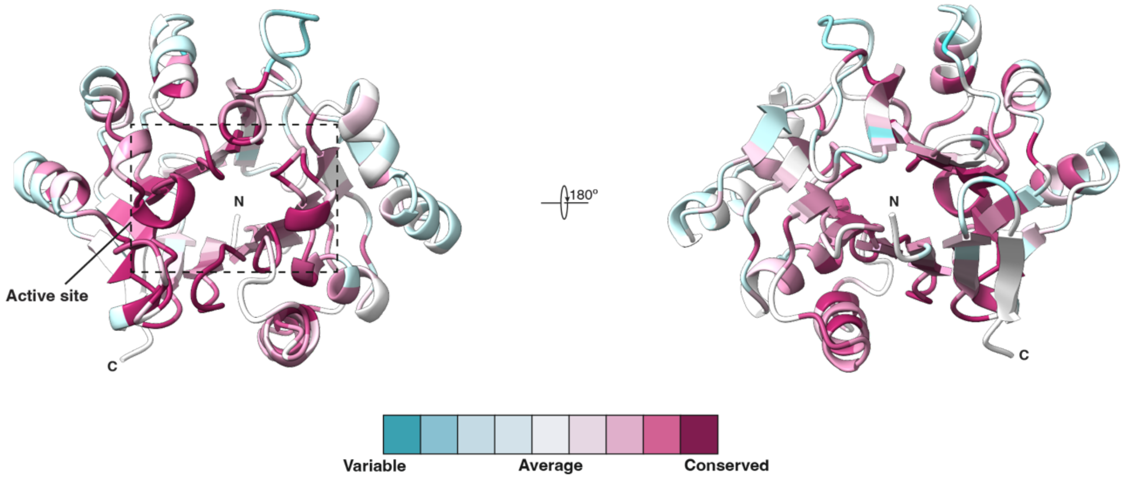

Fig. S4: ConSurf analysis of $BLACT_{Mg}$
Two orientations of $BLACT_{Mg}$ are shown in ribbon representation with residues coloured according to their conservation (cyan, low conservation; purple, high conservation). The active site of $BLACT_{Mg}$ is indicated by a dashed box (left) and is highly conserved. This figure was generated using the ConSurf server (7).

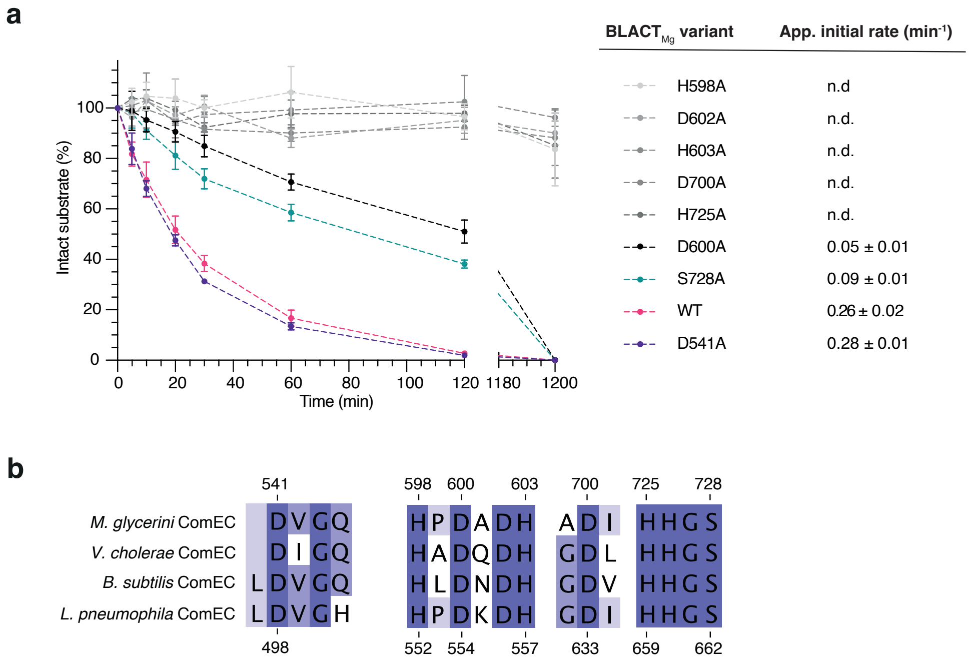

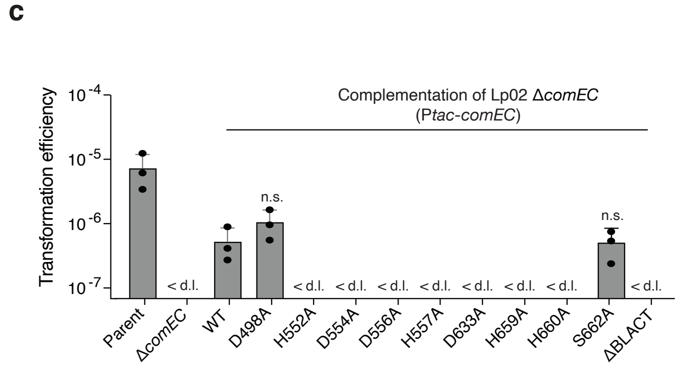

d

Metal coordinating residues

BBL numbering	*M. glycerini*	*L. pneumophila*
H116	H598	H552
H118	D600	D554
D120	D602	D556
H121	H603	H557
D221	D700	D633
H263	H726	H660

Fig. S5: BLACT$_{Mg}$ nuclease activity is essential for natural transformation
a Time-course nuclease activity assay monitoring the degradation of ssDNA by BLACT$_{Mg}$ variants. Nuclease assays were conducted with 1 μM wild-type BLACT$_{Mg}$ and 10 μM ssDNA substrates, 50 nucleotides (nt) in length. DNA substrates were fluorescently labelled with fluorescein (FAM) on the first base (thymine, T) at the 5' terminus. Error bars represent the standard error from three technical replicates. WT, wild type. **b** Multiple sequence alignment of the active site of BLACT$_{Mg}$ from *M. glycerini, Vibrio cholerae, B. subtilis and Legionella pneumophila*. Conservation is indicated by different shades of blue. **c** Transformation efficiencies of parental Lp02, Lp02 *ΔcomEC*, Lp02 *ΔBLACT* and Lp02 *ΔcomEC* strains complemented by ectopic expression of wild-type and mutant versions of ComEC corresponding to the mutations tested in panel **a**. Mean transformation efficiencies of three independent biological replicates are shown with error bars representing the standard deviation (SD). < d.l., below detection limit (d.l.). Statistical differences were determined on log-transformed data using an unpaired two-sided t-test with Welch's correction (8). The Lp02 strain complemented with wild-type ComEC was compared to those strains complemented with ComEC mutants. n.s., not statistically significant. **d** Table with metal coordinating residues according to the class B β-lactamases (BBL) numbering (9) and the corresponding residues in *M. glycerini* and *L. pneumophila*.

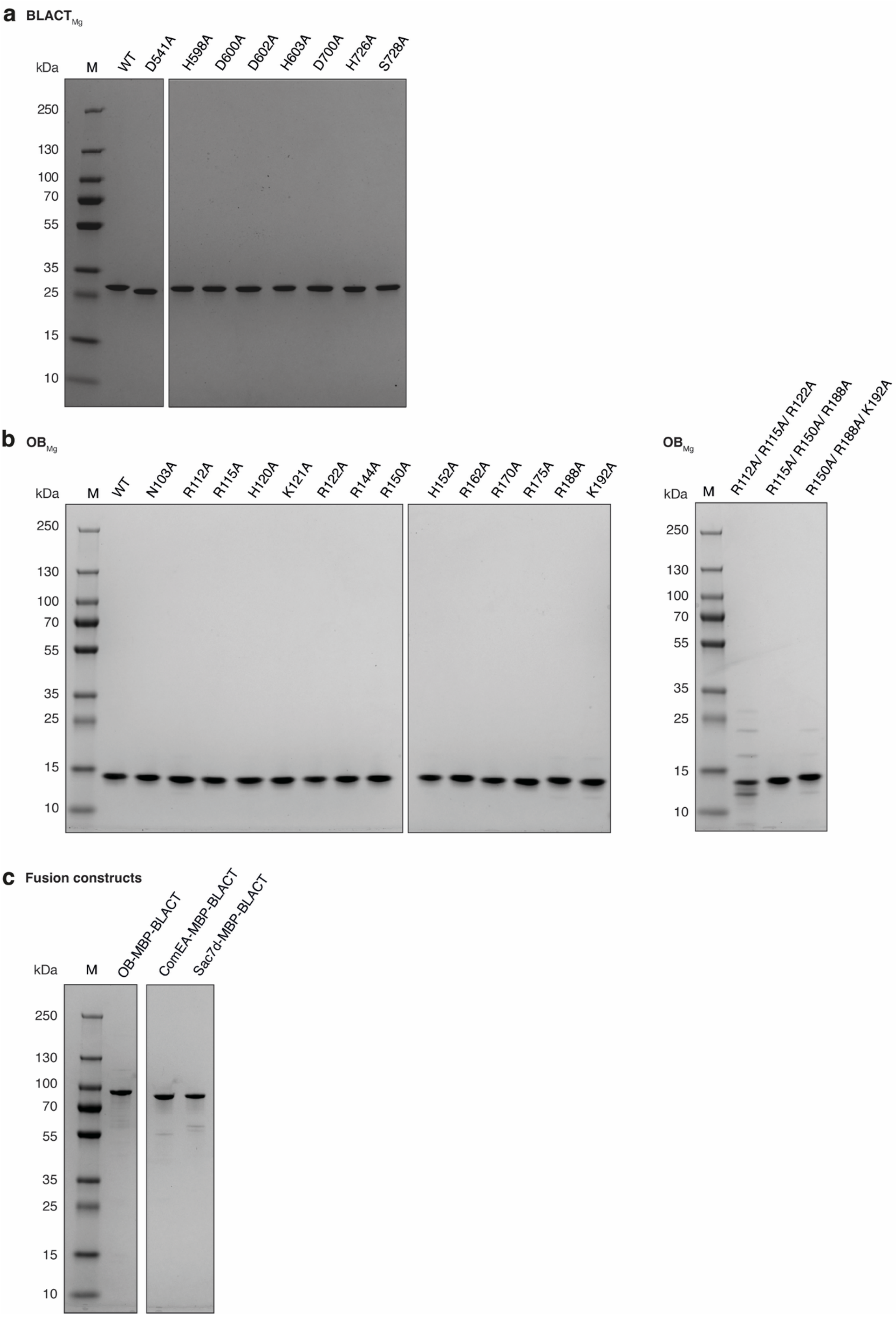

a BLACT$_{Mg}$
kDa
M
WT
D541A
H598A
D600A
D602A
H603A
D700A
H726A
S728A
250
130
100
70
55
35
25
15
10
b OB$_{Mg}$
WT
N103A
R112A
R115A
H120A
K121A
R122A
R144A
R150A
H152A
R162A
R170A
R175A
R188A
K192A
R112A/ R115A/ R122A
R115A/ R150A/ R188A
R150A/ R188A/ K192A
c Fusion constructs
OB-MBP-BLACT
ComEA-MBP-BLACT
Sac7d-MBP-BLACT

Fig. S6: Purified proteins used in this study

a-c Purified $BLACT_{Mg}$ (**a**), OB fold proteins from *M. glycerini* (OB_{Mg}) (**b**), and fusion constructs (**c**), resolved by SDS-PAGE. The OB, ComEA and BLACT domains within the fusion constructs are derived from *M. glycerini*, and Sac7d originates from *Sulfolobus acidocaldarius.* Further construct information can be found in **Table S4**.

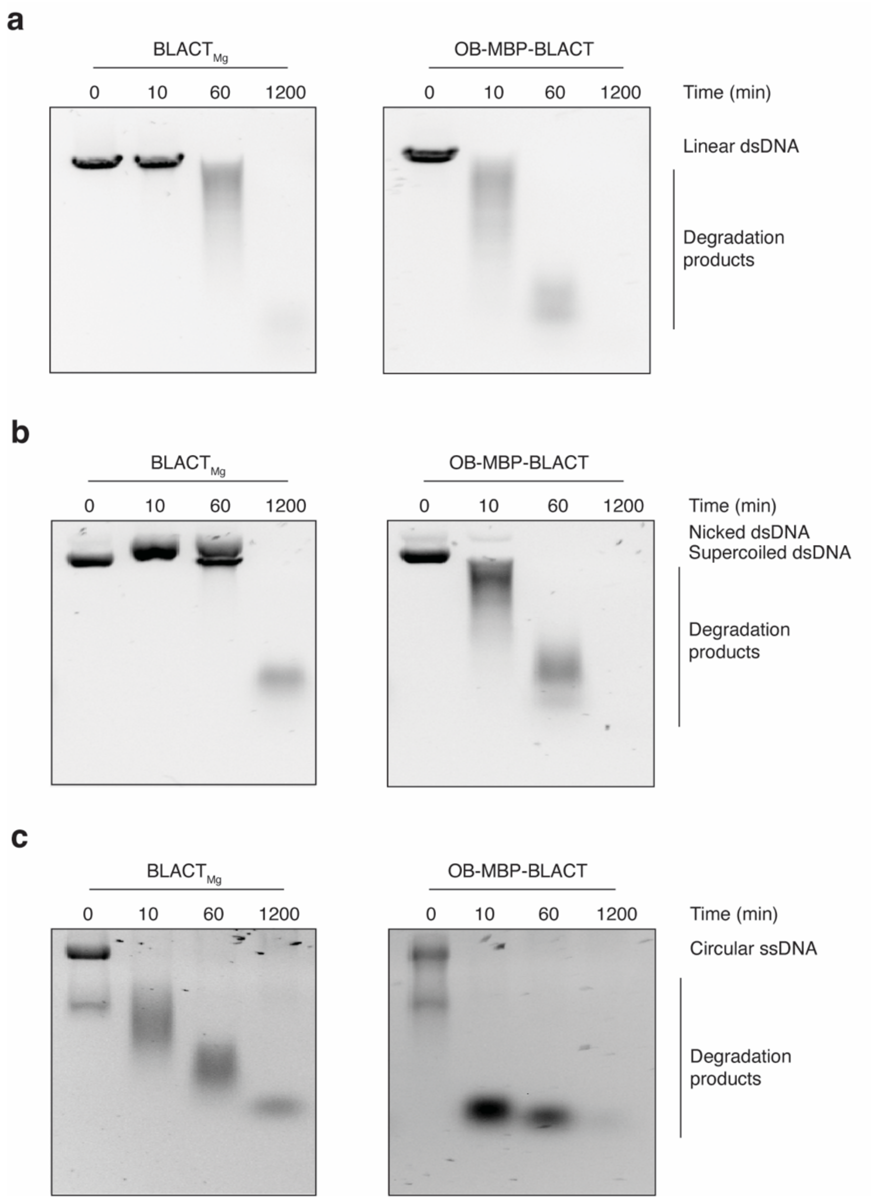

Fig. S7: BLACT$_{Mg}$ and OB-MBP-BLACT degrade circular and linearised plasmid DNA *in vitro*

a-c Time course nuclease assay resolved on 1% (w/v) agarose gels showing the BLACT$_{Mg}$ or OB-MBP-BLACT-dependent degradation of linearised dsDNA (**a**), circular dsDNA (**b**) and circular ssDNA (**c**). All substrate concentrations were adjusted such that the number of cleavable bonds were identical to the number of bonds in Figure 1c. The DNA sequence in (**a**) and (**b**) is identical. Different DNA species are labelled to the right of each gel.

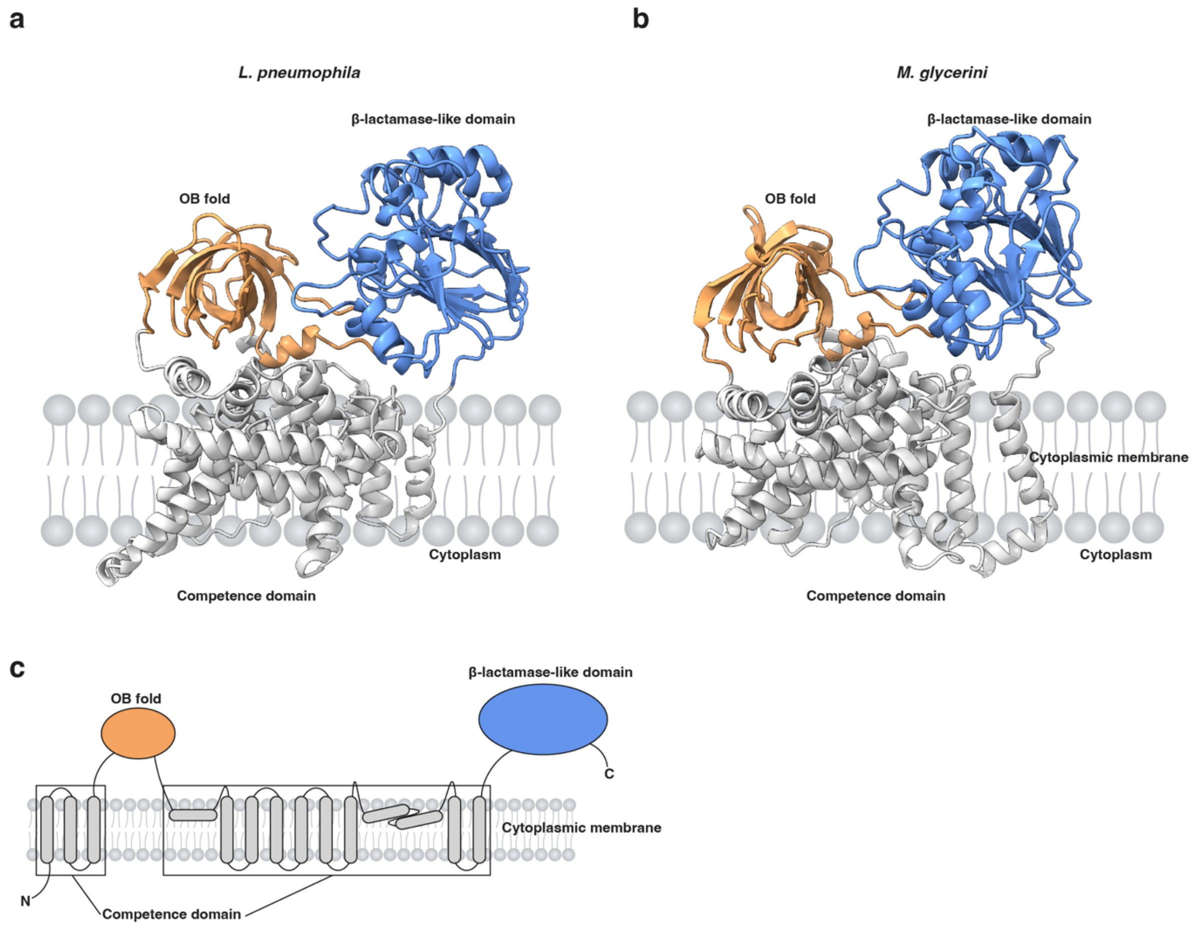

Fig. S8: Predicted three-dimensional domain organisation of full-length ComEC
a, b Structural predictions of *L. pneumophila* (**a**) and *M. glycerini* (**b**) ComEC orthologues using AlphaFold3 (6), shown in ribbon representation (OB fold, orange; β-lactamase-like domain, blue; competence domain, grey). **c** Schematic domain architecture of ComEC. The domains are coloured as in panels (**a**) and (**b**).

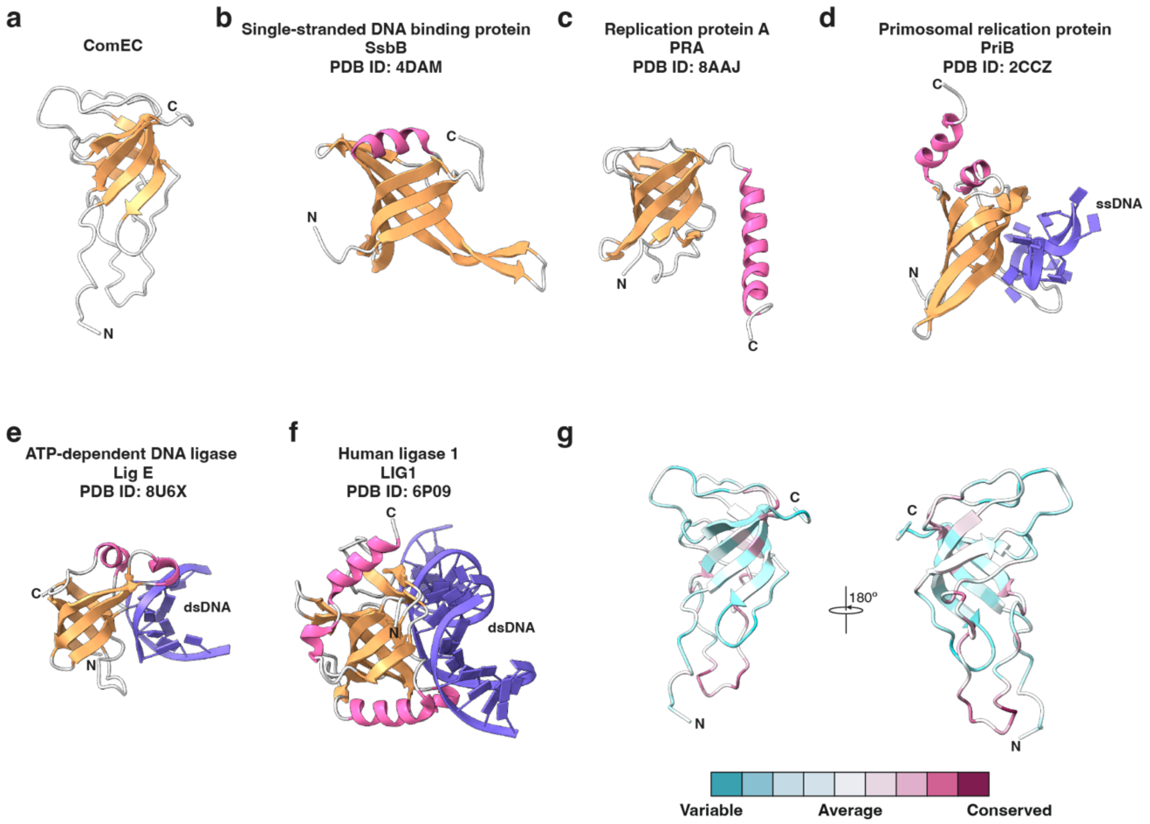

Fig. S9: Structures of OB fold-containing proteins and ConSurf analysis of OB_{Mg}
a-f Structural comparison of OB folds from different proteins, including the OB fold from $ComEC_{Mg}$ (this study) (**a**), the single-stranded DNA binding protein (SsbB) from *S. coelicolor* (PDB ID: 4DAM) (10) (**b**), the OB-4 domain of the replication protein A (RPA) complex from *Pyrococcus abyssi* (PDB ID: 8AAJ) (11) (**c**), the primosomal replication protein B (PriB) from *Escherichia coli* bound to ssDNA (PDB ID: 2CCZ) (12) (**d**), the OB domain of ATP-dependent DNA ligase (Lig E) from *Neisseria gonorrhoeae* bound to dsDNA (PDB ID: 8U6X) (13) (**e**), and the OB domain of human ligase 1 (LIG1) bound to dsDNA (PDB ID: 6P09) (14) (**f**). Structures are shown in ribbon representation (β-strands, orange; α-helices, pink; loops, grey; ssDNA and dsDNA, purple). **g** Two orientations of OB_{Mg} with residues coloured according to conservation as in **Fig. S4**. This panel was generated with the ConSurf server (7).

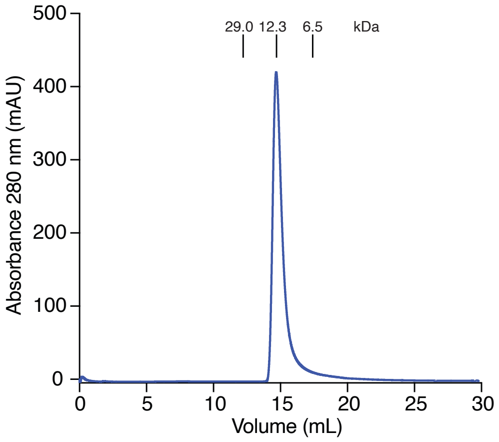

Fig. S10: OB_{Mg} is a monomer in solution
Analytical size exclusion chromatography showing a single, monodisperse peak for OB_{Mg} (14 kDa) eluting at 15 mL. Molecular weights of globular proteins and their corresponding elution volumes, according to the manufacturer, are indicated above the chromatogram.

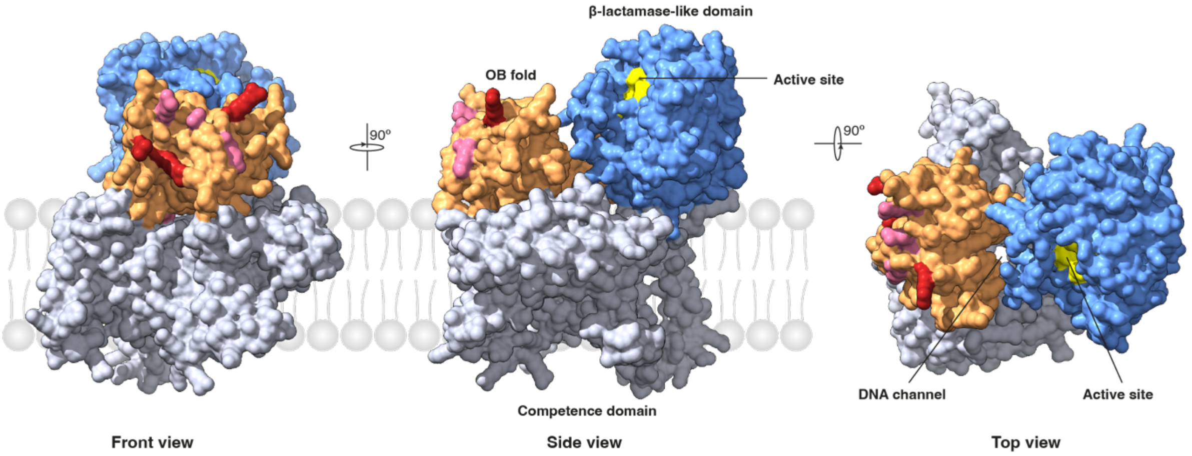

Fig. S11: DNA binding residues are solvent exposed on the predicted structure of full-length ComEC

The front, side and top view of full-length ComEC from *M. glycerini* ($ComEC_{Mg}$) predicted by AlphaFold3 (6) shown in surface representation (OB fold, orange; β-lactamase-like domain, blue; competence domain, grey). DNA binding residues identified in **Fig. 3d, e** coloured in shades of red. The active site of the β-lactamase-like domain and the DNA channel are also indicated on the figure.

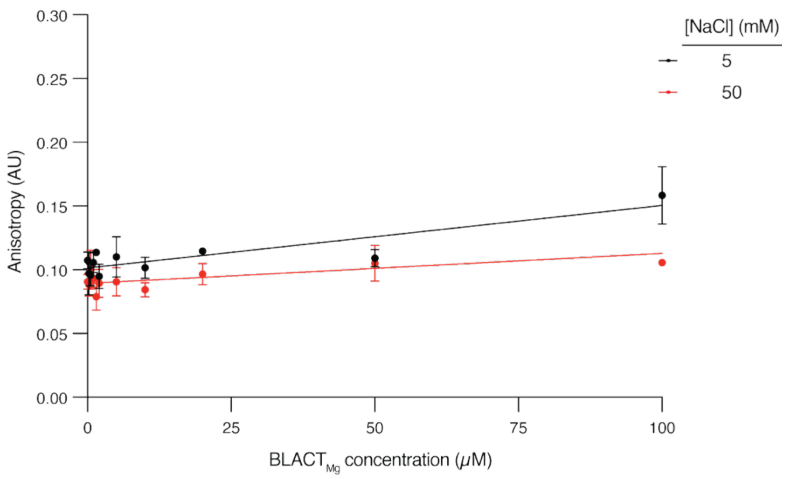

Fig. S12: BLACT$_{Mg}$ does not bind DNA
Fluorescence anisotropy binding experiment testing the interaction between 12 bp FAM-labelled dsDNA containing nuclease-resistant phosphorothioate (PTO) bonds and BLACT$_{Mg}$. The experiment was conducted in buffer containing 5 mM and 50 mM NaCl. Each experiment was performed in triplicate and error bars represent the standard error of the mean.

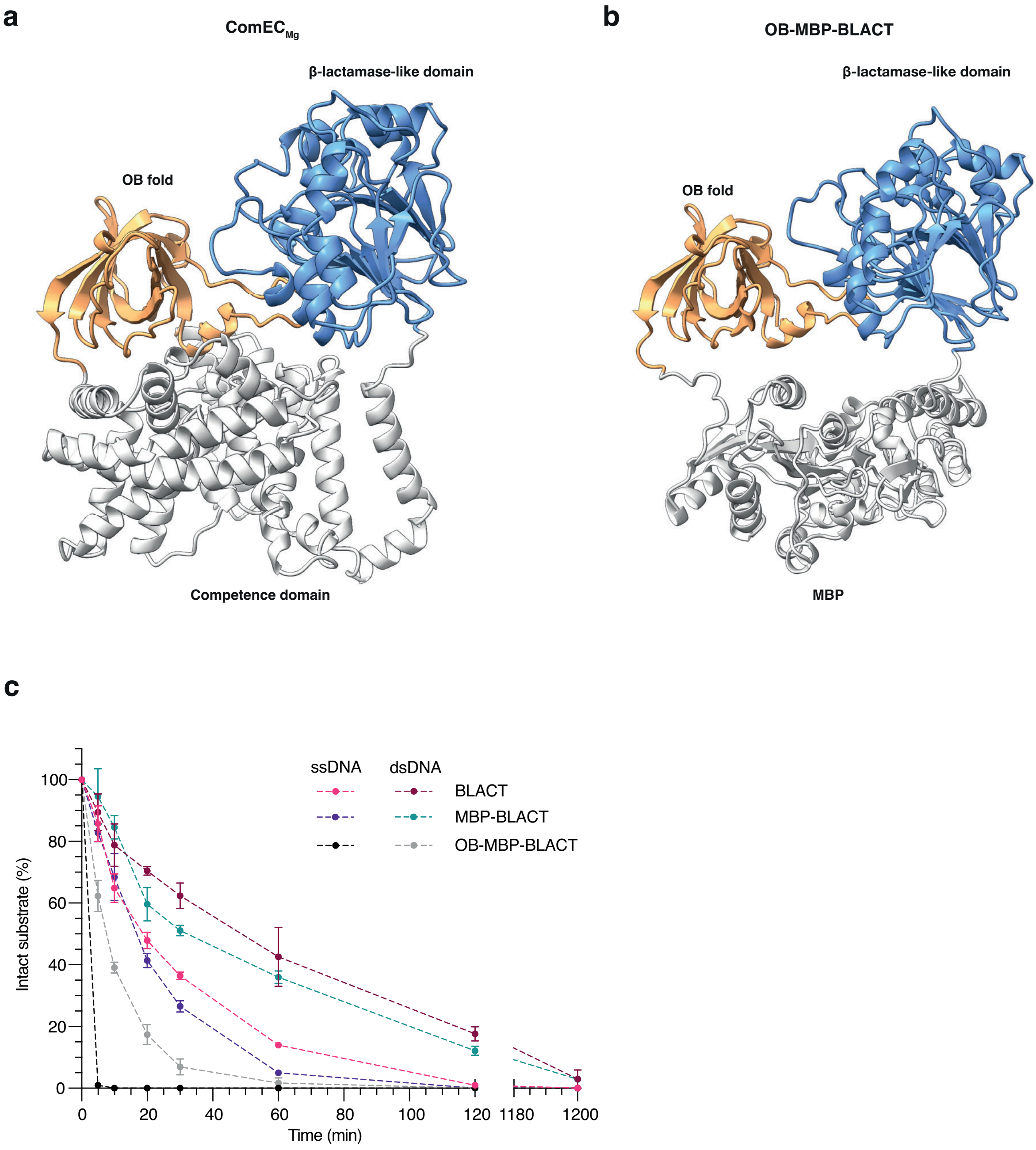

Fig. S13: OB-MBP-BLACT displays a similar predicted domain organisation as ComEC
a, b Full-length ComEC$_{Mg}$ (**a**) and OB-MBP-BLACT (**b**) predicted by AlphaFold3 (6) shown in ribbon representation (OB fold, orange; β-lactamase-like domain, blue; competence domain/MBP, grey). **c** Time-course nuclease activity assay monitoring the degradation of ssDNA and dsDNA by BLACT$_{Mg}$, MBP-BLACT and OB-MBP-BLACT constructs. Nuclease assays were conducted with 1 µM enzyme and 10 µM ssDNA or 5 µM dsDNA substrates, 50 nucleotides (nt) in length. DNA substrates were fluorescently labelled with fluorescein (FAM) on the first base (thymine, T) at the 5' terminus. Error bars represent the standard error derived from three technical replicates.

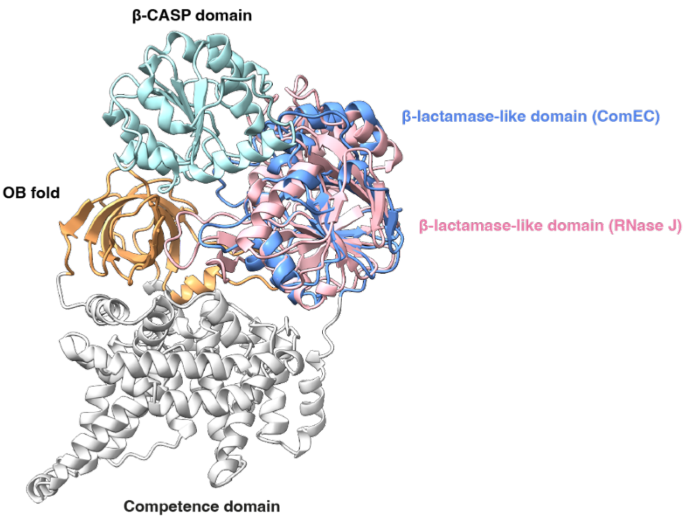

Fig. S14: The relative positioning of the OB fold and β-CASP domains in ComEC and RNase J

Superposition of RNase J from *S. coelicolor* (PDB ID: 5A0T) (1) and $ComEC_{Mg}$ using the β-lactamase-like domain to align the structures. Structures are displayed in ribbon representation (OB fold, orange; competence domain, grey; β-CASP domain, cyan; β-lactamase-like domain from $ComEC_{Mg}$, blue; β-lactamase-like domain from RNase J, pink).

Supplementary Table 1: Data collection and refinement statistics of $BLACT_{Mg}$

**Protein**	$BLACT_{Mg}$
**Data collection**	
Wavelength (Å)	0.999989
Space group	$C222_1$
Cell dimensions	
a, b, c (Å)	80.957, 96.849, 71.719
α, β, γ (°)	90, 90, 90
Resolution (Å)	62.11-1.86 (1.90-1.86)
R_{sym} or R_{merge}	0.069(0.352)
Completeness (%)	99.52(99.16)
Redundancy	6.6(6.9)
Wilson B-factor	22.46
**Refinement**	
Resolution (Å)	62.11-1.86 (1.90-1.86)
Unique reflections	23812
R_{work} / R_{free}	0.1696/0.2027
No. atoms (non-hydrogen)	
Protein	2064
Ligand	5
Water	138
Protein residues	266
Average B-factor	29.39
B-factors	
Protein	29.07
Ligand	50.18
Water	33.37
R.m.s deviations	
Bond lengths (Å)	0.004
Bond angles (°)	0.79
Ramachandran plot	
Favored (%)	95.83
Allowed (%)	4.17
Outliers (%)	0
Rotamer outliers (%)	0.91
Clashscore	1.46
**PDB code**	9IC4

Single crystal is used for the diffraction measurement.
Numbers in brackets are for the highest resolution shell.

Supplementary Table 2: NMR structural statistics of OB_{Mg}

Protein	OB_{Mg}
**NMR distance and dihedral constraints**	
Distance constraints	
Total NOE	2098
Intra-residue	379(18.1%)
Inter-residue	
Sequential ($\|i-j\| = 1$)	887(42.3%)
Medium-range ($\|i-j\| < 5$)	214(10.2%)
Long-range ($\|i-j\| \geq 5$)	997(47.5%)
Hydrogen bonds	0
Total dihedral angle restraints	140
ϕ	70
ψ	70
**Structure statistics***	
Violations (mean)	
Distance constraints (Å)	2(0.20)
Dihedral angle constraints (º)	4 (21)
Max. dihedral angle violation (º)	22
Max. distance constraint violation (Å)	0.39
Deviations from idealized geometry	
Bond lengths (Å)	0.0036 ± 0.0012
Bond angles (º)	1.482 ± 0.494
Average pairwise r.m.s. deviation** (Å)	
Heavy	0.49 ± 0.11
Backbone	0.85 ± 0.14

* Statistics computed for the deposited bundle of 20 violation energy best structures selected out of 30 amber energy best structures.

** Pairwise r.m.s. deviation for the structured regions comprising residues 86–155 and 182–199 was calculated among 20 out of 30 refined structures.

Supplementary Table 3: Bacterial strains used in this study

Name	Relevant genotype/description	Source/Reference
E. coli		
BL21 (DE3)	*E. coli* expression strain	NEB (cat. no. C2527H/I)
Stellar (HST08 strain)	*E. coli* cloning strain	Takara (cat. no. 636763/636766)
DH5α λpir	*E. coli cloning strain:* Encodes π protein for the replication of the *pir*-dependent origin of replication – *oriR* (*R6K*)	(15, 16)
L. pneumophila		
Lp02 WT	Philadelphia-1 *rpsL hsdR thyA*; SmR	(17)
Lp02 *ΔcomEC*	Lp02 Δ*comEC*	(18)

Supplementary Table 4: Plasmids used in this study

Name	Relevant genotype/description	Source/Reference
pMMB207C	*Legionella* expression vector derived from RSF1010: IncQ lacIq *Ptac oriT* Δ*mobA*; CmR	(19)
pMMB207C-*comEC$_{Lp}$*	*L. pneumophila* wild-type *comEC*	This study
pMMB207C-*comEC$_{Lp}$* D498A	pMMB207C-*comEC$_{Lp}$*, with *comEC* D498A mutation	This study
pMMB207C-*comEC$_{Lp}$* H552A	pMMB207C-*comEC$_{Lp}$*, with *comEC* H552A mutation	This study
pMMB207C-*comEC$_{Lp}$* D554A	pMMB207C-*comEC$_{Lp}$*, with *comEC* D554A mutation	This study
pMMB207C-*comEC$_{Lp}$* D556A	pMMB207C-*comEC$_{Lp}$*, with *comEC* D556A mutation	This study
pMMB207C-*comEC$_{Lp}$* H557A	pMMB207C-*comEC$_{Lp}$*, with *comEC* *H*557A mutation	This study
pMMB207C-*comEC$_{Lp}$* D663A	pMMB207C-*comEC$_{Lp}$*, with *comEC* D663A mutation	This study
pMMB207C-*comEC$_{Lp}$* H659A	pMMB207C-*comEC$_{Lp}$*, with *comEC* H659A mutation	This study
pMMB207C-*comEC$_{Lp}$* H660A	pMMB207C-*comEC$_{Lp}$*, with *comEC* H660A mutation	This study
pMMB207C-*comEC$_{Lp}$* S662A	pMMB207C-*comEC$_{Lp}$*, with *comEC* S662A mutation	This study
pMMB207C-*comEC$_{Lp}$* ΔBLACT	pMMB207C-*comEC$_{Lp}$*, with *comEC* BLACT deletion (Δ489-735)	This study
pMMB207C-*comEC$_{Lp}$* F523A	pMMB207C-*comEC$_{Lp}$*, with *comEC* F523A mutation	This study
pMMB207C-*comEC$_{Lp}$* Y522A/F523A	pMMB207C-*comEC$_{Lp}$*, with *comEC* Y522A/F523A mutation	This study
pMMB207C-*comEC$_{Lp}$* R688A	pMMB207C-*comEC$_{Lp}$*, with *comEC* R688A mutation	This study
pMMB207C-*comEC$_{Lp}$* F689A	pMMB207C-*comEC$_{Lp}$*, with *comEC* F689A mutation	This study
pMMB207C-*comEC$_{Lp}$* F691A	pMMB207C-*comEC$_{Lp}$*, with *comEC* F691A mutation	This study
pMMB207C-*comEC$_{Lp}$* F689A/F691A	pMMB207C-*comEC$_{Lp}$*, with *comEC* F689A/F691A mutation	This study
pMMB207C-*comEC$_{Lp}$* Y108A	pMMB207C-*comEC$_{Lp}$*, with *comEC* Y108A mutation	This study
pMMB207C-*comEC$_{Lp}$* Y140A	pMMB207C-*comEC$_{Lp}$*, with *comEC* Y140A mutation	This study
pMMB207C-*comEC$_{Lp}$* Y154A	pMMB207C-*comEC$_{Lp}$*, with *comEC* Y154A mutation	This study
pMMB207C-*comEC$_{Lp}$* W212A	pMMB207C-*comEC$_{Lp}$*, with *comEC* W212A mutation	This study
pMMB207C-*comEC$_{Lp}$* F331A	pMMB207C-*comEC$_{Lp}$*, with *comEC* F331A mutation	This study
pMMB207C-*comEC$_{Lp}$* Y373A	pMMB207C-*comEC$_{Lp}$*, with *comEC* *Y373*A mutation	This study
pOPINS	*E. coli* expression vector, N-terminal His_6-SUMO tag, T7 promoter, KanR	(20)
pOPINS-*BLACT$_{Mg}$*	*M. glycerini* β-lactamase-like domain (532-797)	This study
pOPINS-*BLACT$_{Mg}$* D541A	pOPINS-*BLACT$_{Mg}$*, with BLACT D541A mutation	This study
pOPINS-*BLACT$_{Mg}$* H598A	pOPINS-*BLACT$_{Mg}$*, with BLACT H598A mutation	This study

pOPINS-*BLACT*$_{Mg}$ D600A	pOPINS-*BLACT*$_{Mg}$, with BLACT D600A mutation	This study
pOPINS-*BLACT*$_{Mg}$ D602A	pOPINS-*BLACT*$_{Mg}$, with BLACT D602A mutation	This study
pOPINS-*BLACT*$_{Mg}$ H603A	pOPINS-*BLACT*$_{Mg}$, with BLACT H603A mutation	This study
pOPINS-*BLACT*$_{Mg}$ D700A	pOPINS-*BLACT*$_{Mg}$, with BLACT D700A mutation	This study
pOPINS-BLACT$_{Mg}$ H725A	pOPINS-*BLACT*$_{Mg}$, with BLACT H725A mutation	This study
pOPINS-*BLACT*$_{Mg}$ S728A	pOPINS-*BLACT*$_{Mg}$, with BLACT S728A mutation	This study
pOPINS-*OB*$_{Mg}$	*M. glycerini* OB fold (76-199)	This study
pOPINS-*OB*$_{Mg}$ N103A	pOPINS-*OB*$_{Mg}$, with N103A mutation	This study
pOPINS-*OB*$_{Mg}$ R112A	pOPINS-*OB*$_{Mg}$, with R112A mutation	This study
pOPINS-*OB*$_{Mg}$ R115A	pOPINS-*OB*$_{Mg}$, with R115A mutation	This study
pOPINS-*OB*$_{Mg}$ H120A	pOPINS-*OB*$_{Mg}$, with H120A mutation	This study
pOPINS-*OB*$_{Mg}$ K121A	pOPINS-*OB*$_{Mg}$, with K121A mutation	This study
pOPINS-*OB*$_{Mg}$ R122A	pOPINS-*OB*$_{Mg}$, with R122A mutation	This study
pOPINS-*OB*$_{Mg}$ R144A	pOPINS-*OB*$_{Mg}$, with R144A mutation	This study
pOPINS-*OB*$_{Mg}$ R150A	pOPINS-*OB*$_{Mg}$, with R150A mutation	This study
pOPINS-*OB*$_{Mg}$ H152A	pOPINS-*OB*$_{Mg}$, with H152A mutation	This study
pOPINS-*OB*$_{Mg}$ R162A	pOPINS-*OB*$_{Mg}$, with R162A mutation	This study
pOPINS-*OB*$_{Mg}$ R170A	pOPINS-*OB*$_{Mg}$, with R170A mutation	This study
pOPINS-*OB*$_{Mg}$ R175A	pOPINS-*OB*$_{Mg}$, with R175A mutation	This study
pOPINS-*OB*$_{Mg}$ R188A	pOPINS-*OB*$_{Mg}$, with R188A mutation	This study
pOPINS-*OB*$_{Mg}$ K192A	pOPINS-*OB*$_{Mg}$, with K192A mutation	This study
pOPINS-*OB*$_{Mg}$ R112A/R115A/R122A	pOPINS-*OB*$_{Mg}$, with R112A, R115A, R122A mutations	This study
pOPINS-*OB*$_{Mg}$ R115A/R150A/R188A	pOPINS-*OB*$_{Mg}$, with R115A, R150A, R188A mutations	This study
pOPINS-*OB*$_{Mg}$ R150A/R188A/K192A	pOPINS-*OB*$_{Mg}$, with R150A, R188A, K192A mutations	This study
pOPINS-*OB-MBP-BLACT*	OB-MBP-BLACT fusion protein (OB and BLACT domains from *M. glycerini*)	This study
pOPINS-*ComEA-MBP-BLACT*	ComEA-MBP-BLACT fusion protein (ComEA and BLACT domains from *M. glycerini*)	This study
pOPINS-*Sac7d-MBP-BLACT*	Sac7d-MBP-BLACT fusion protein (Sac7d from *S. acidocaldarius* and BLACT from *M. glycerini*)	This study
pTRC99A-*lpg2953-2958::Kan*	*L. pneumophila* genomic region spanning *lpg2953-2958*. The *hipB* gene (*lpg2955*) is disrupted by the kanamycin resistance gene, Kan	(18)

Supplementary Table 5: Oligonucleotides used in this study

Name	Sequence (5' to 3')	Construct
Cloning		
Lpg2953-Fw	ATCTCTGGTGTGTTCGGATAGATTATGCGAGAGGTCTATTTGAAGATTCTCTGACTATG	pTRC99A-*lpg2953-2958::Kan* amplification of tDNA for transformation assay
Lpg2958-Rv	GTCGACTCTAGACACAGACATGGCCTGGAAACGTTGGTGGG	pTRC99A-*lpg2953-2958::Kan* amplification of tDNA for transformation assay
pBR322-Fw	CGACCGAGTTGCTCTTGCCCG	linearised pBR322 for nuclease activity assays
pBR322-Rv	CCGCATACACTATTCTCAGAATGACTTGGTTGAG	linearised pBR322 for nuclease activity assays
pMMB207C-*comEC$_{Lp}$*-ΔBLACT-Fw	CACCTCGTACTGCAATTTATTAAATGGATTGGCTGACCCATGTTATATCTAAGCC	pMMB207C-*comEC$_{Lp}$*-ΔBLACT
pMMB207C-*comEC$_{Lp}$*-ΔBLACT-Rv	ATAAATTGCAGTACGAGGTGGAAATAAGGGAAGG	pMMB207C-*comEC$_{Lp}$*-ΔBLACT
pMMB207C-*comEC$_{Lp}$*-D498A-Fw	AATATTTTGGCTGTTGGGCAAGGACTTG	pMMB207C-*comEC$_{Lp}$*-D498A
pMMB207C-*comEC$_{Lp}$*-D498A-Rv	AATATTTTGGCTGTTGGGCAAGGACTTG	pMMB207C-*comEC$_{Lp}$*-D498A
pMMB207C-*comEC$_{Lp}$*-H552A-Fw	GTAATAAGCGCTCCCGATAAAGATCATAAAGGCG	pMMB207C-*comEC$_{Lp}$*-H552A
pMMB207C-*comEC$_{Lp}$*-H552A-Rv	TTATCGGGAGCGCTTATTACTATTTTGTCAATTGTTTTTATTCC	pMMB207C-*comEC$_{Lp}$*-H552A
pMMB207C-*comEC$_{Lp}$*-D554A-Fw	CATCCCGCTAAAGATCATAAAGGCGG	pMMB207C-*comEC$_{Lp}$*-D554A
pMMB207C-*comEC$_{Lp}$*-D554A-Rv	TATGATCTTTAGCGGGATGGCTTATTACTATTTTGTCAATTG	pMMB207C-*comEC$_{Lp}$*-D554A
pMMB207C-*comEC$_{Lp}$*-D556A-Fw	CCGATAAAGCTCATAAAGGCGGATTAAATTCCC	pMMB207C-*comEC$_{Lp}$*-D556A
pMMB207C-*comEC$_{Lp}$*-D556A-Rv	GCCTTTATGAGCTTTATCGGGATGGCTTATTACTATTTTG	pMMB207C-*comEC$_{Lp}$*-D556A
pMMB207C-*comEC$_{Lp}$*-H557A-Fw	CCGATAAAGATGCTAAAGGCGGATTAAATTCCC	pMMB207C-*comEC$_{Lp}$*-H557A
pMMB207C-*comEC$_{Lp}$*-H557A-Rv	GCCTTTAGCATCTTTATCGGGATGGCTTATTACTATTTTG	pMMB207C-*comEC$_{Lp}$*-H557A
pMMB207C-*comEC$_{Lp}$*-D663A-Fw	AACGGGAGCTATTGAAAAAGCAGCTGAAGAGTAC	pMMB207C-*comEC$_{Lp}$*-D663A
pMMB207C-*comEC$_{Lp}$*-D663A-Rv	GCTTTTTCAATAGCTCCCGTTAACAAAACCCTGC	pMMB207C-*comEC$_{Lp}$*-D663A
pMMB207C-*comEC$_{Lp}$*-H659A-Fw	GTGCCTGCTCATGGGAGCAAAACGTC	pMMB207C-*comEC$_{Lp}$*-H659A
pMMB207C-*comEC$_{Lp}$*-H659A-Rv	GCTCCCATGAGCAGGCACAACTAAAA	pMMB207C-*comEC$_{Lp}$*-H659A
pMMB207C-*comEC$_{Lp}$*-H660A-Fw	GCCTCATGCTGGGAGCAAAACGTCTTC	pMMB207C-*comEC$_{Lp}$*-H660A
pMMB207C-*comEC$_{Lp}$*-H660A-Rv	GCTCCCAGCATGAGGCACAACTAAAATTTCGGATGCCAG	pMMB207C-*comEC$_{Lp}$*-H660A
pMMB207C-*comEC$_{Lp}$*-S662A-Fw	CATCATGGGGCTAAAACGTCTTCATCC	pMMB207C-*comEC$_{Lp}$*-S662A

pMMB207C-$comEC_{Lp}$-S662A-Rv	GACGTTTTAGCCCCATGATGAGGCAC	pMMB207C-$comEC_{Lp}$-S662A
pMMB207C-$comEC_{Lp}$-F523A-Fw	TTCCTACGCGCAGGGGAGTGATTTAGGGCAAATGGC	pMMB207C-$comEC_{Lp}$-F523A
pMMB207C-$comEC_{Lp}$-F523A-Rv	TCCCCTGCGCGTAGGAATCTCCCGTGTCGTAGAGTAAAAC	pMMB207C-$comEC_{Lp}$-F523A
pMMB207C-$comEC_{Lp}$-Y522A/F523A-Fw	AGATTCCGCGGCGCAGGGGAGTGATTTAGGGCAAATGGC	pMMB207C-$comEC_{Lp}$-Y522A/F523A
pMMB207C-$comEC_{Lp}$-Y522A/F523A-Rv	TCCCCTGCGCCGCGGAATCTCCCGTGTCGTAGAGTAAAACATG	pMMB207C-$comEC_{Lp}$-Y522A/F523A
pMMB207C-$comEC_{Lp}$-R688A-Fw	TGATAATGCGTTCAAATTTCCACATTCTAAAACTTTGCAAAGCATG	pMMB207C-$comEC_{Lp}$-R688A
pMMB207C-$comEC_{Lp}$-R688A-Rv	ATTTGAACGCATTATCAAATCCTAAGGATGCAATGGCATAAAGC	pMMB207C-$comEC_{Lp}$-R688A
pMMB207C-$comEC_{Lp}$-F689A-Fw	TAATCGCGCGAAATTTCCACATTCTAAAACTTTGCAAAGCATGAAAAC	pMMB207C-$comEC_{Lp}$-F689A
pMMB207C-$comEC_{Lp}$-F689A-Rv	GAAATTTCGCGCGATTATCAAATCCTAAGGATGCAATGGC	pMMB207C-$comEC_{Lp}$-F689A
pMMB207C-$comEC_{Lp}$-F691A-Fw	CTTCAAAGCGCCACATTCTAAAACTTTGCAAAGCATGAAAACATTAG	pMMB207C-$comEC_{Lp}$-F691A
pMMB207C-$comEC_{Lp}$-F691A-Rv	AATGTGGCGCTTTGAAGCGATTATCAAATCCTAAGGATGCAATG	pMMB207C-$comEC_{Lp}$-F691A
pMMB207C-$comEC_{Lp}$-F689A/F691A-Fw	TTGATAATCGCGCGAAAGCGCCACATTCTAAAACTTTGCAAAGCATGAAAACATTAG	pMMB207C-$comEC_{Lp}$-F689A/F691A
pMMB207C-$comEC_{Lp}$-F689A/F691A-Rv	TTAGAATGTGGCGCTTTCGCGCGATTATCAAATCCTAAGGATGCAATGGC	pMMB207C-$comEC_{Lp}$-F689A/F691A
pMMB207C-$comEC_{Lp}$-Y108A-Fw	AAACTGGGCGAACAAACCACCTGTCTTACGAGCC	pMMB207C-$comEC_{Lp}$-Y108A
pMMB207C-$comEC_{Lp}$-Y108A-Rv	GTTTGTTCGCCCAGTTTAATTGGATCAAGCCTTGAGCAG	pMMB207C-$comEC_{Lp}$-Y108A
pMMB207C-$comEC_{Lp}$-Y140A-Fw	TTTTAATGCGGTACGTTACCTGGCGGCTCG	pMMB207C-$comEC_{Lp}$-Y140A
pMMB207C-$comEC_{Lp}$-Y140A-Rv	AACGTACCGCATTAAAACCTCCCGGGTTGTGGAAG	pMMB207C-$comEC_{Lp}$-Y140A
pMMB207C-$comEC_{Lp}$-Y154A-Fw	GACTGGCGCGATTCGTTATCGGAATAATAAGCTAATTAGCAATCAACC	pMMB207C-$comEC_{Lp}$-Y154A
pMMB207C-$comEC_{Lp}$-Y154A-Rv	AACGAATCGCGCCAGTCCAATGGATATGACGAGC	pMMB207C-$comEC_{Lp}$-Y154A
pMMB207C-$comEC_{Lp}$-W212A-Fw	AGATCATGCGAATTTGTTCCGAAGAACAGGAACGATAC	pMMB207C-$comEC_{Lp}$-W212A
pMMB207C-$comEC_{Lp}$-W212A-Rv	ACAAATTCGCATGATCTGAACTGATATGGTGAGTAACATTTAACGTC	pMMB207C-$comEC_{Lp}$-W212A
pMMB207C-$comEC_{Lp}$-F331A-Fw	TTTTTACGCGTCATTTCTTGCCGTGGCCTGTC	pMMB207C-$comEC_{Lp}$-F331A
pMMB207C-$comEC_{Lp}$-F331A-Rv	GAAATGACGCGTAAAAACCTTGCAGGAATACGGCATG	pMMB207C-$comEC_{Lp}$-F331A
pMMB207C-$comEC_{Lp}$-Y373A-Fw	TTTTTACGCGTCATTTCTTGCCGTGGCCTGTC	pMMB207C-$comEC_{Lp}$-Y373A
pMMB207C-$comEC_{Lp}$-Y373A-Rv	GAAATGACGCGTAAAAACCTTGCAGGAATACGGCATG	pMMB207C-$comEC_{Lp}$-Y373A
pOPINS-linearise-Fw	TAAAGCTTTCTAGACCATTTAAACACCACCACCACC	pOPINS fragment

pOPINS-linearise-Rv	ACCACCGATCTGTTCGCGATGCG	pOPINS fragment
pOPINS-*BLACT*$_{Mg}$-Fw	GCGAACAGATCGGTGGTCAAGAAGAGCTGAAAGTCACTTTCATCGATGTTGG	pOPINS-*BLACT*$_{Mg}$
pOPINS-*BLACT*$_{Mg}$-Rv	ATGGTCTAGAAAGCTTTAAATCGTTGTGTTGACTTGCCAACGCTC	pOPINS-*BLACT*$_{Mg}$
pOPINS-*BLACT*$_{Mg}$-D541A-Fw	GCGAACAGATCGGTGGTCAAGAAGAGCTGAAAGTCACTTTCATCGCGGTTGG	pOPINS-*BLACT*$_{Mg}$ D541A
pOPINS-*BLACT*$_{Mg}$-D541A-Rv	ATGGTCTAGAAAGCTTTAAATCGTTGTGTTGACTTGCCAACGCTC	pOPINS-*BLACT*$_{Mg}$ D541A
pOPINS-*BLACT*$_{Mg}$-H598A-Fw	AGCACGGCGCCGGACGCGGACCACATCGG	pOPINS-*BLACT*$_{Mg}$ H598A
pOPINS-*BLACT*$_{Mg}$-H598A-Rv	GTCCGGCGCCGTGCTAACAACAACGTCTAAATGGCG	pOPINS-*BLACT*$_{Mg}$ H598A
pOPINS-*BLACT*$_{Mg}$-D600A-Fw	GTTAGCACGCATCCGGCGGCGGACCACATC	pOPINS-*BLACT*$_{Mg}$ D600A
pOPINS-*BLACT*$_{Mg}$-D600A-Rv	CTGCCAGACCACCGATGTGGTCCGCCGC	pOPINS-*BLACT*$_{Mg}$ D600A
pOPINS-*BLACT*$_{Mg}$-D602A-Fw	GACGCGGCGCACATCGGTGGTCTGGCAGCG	pOPINS-*BLACT*$_{Mg}$ D602A
pOPINS-*BLACT*$_{Mg}$-D602A-Rv	GATGTGCGCCGCGTCCGGATGCGTGCTAAC	pOPINS-*BLACT*$_{Mg}$ D602A
pOPINS-*BLACT*$_{Mg}$-H603A-Fw	GCGGACGCGATCGGTGGTCTGGCAGCGGTTG	pOPINS-*BLACT*$_{Mg}$ D603A
pOPINS-*BLACT*$_{Mg}$-H603A-Rv	ACCGATCGCGTCCGCGTCCGGATGCGTGC	pOPINS-*BLACT*$_{Mg}$ D603A
pOPINS-*BLACT*$_{Mg}$-D700A-Fw	GTCTGCTGTTGTCCGCTGCGATTGAAGCTGAG	pOPINS-*BLACT*$_{Mg}$ D700A
pOPINS-*BLACT*$_{Mg}$-D700A-Rv	CTTTTAAGTCTGCCATTGCCTCAGCTTCAATCGC	pOPINS-*BLACT*$_{Mg}$ D700A
pOPINS-*BLACT*$_{Mg}$-H725A-Fw	GCGTTCGACTGTATTTAAGGTACCAGCGCATGG	pOPINS-*BLACT*$_{Mg}$ H725A
pOPINS-*BLACT*$_{Mg}$-H725A-Rv	CAAGACCATAACGGGACCCATGCGCTGGTACC	pOPINS-*BLACT*$_{Mg}$ H725A
pOPINS-*BLACT*$_{Mg}$-S728A-Fw	GTATTTAAGGTACCACATCATGGGGCGCGTTATGGTC	pOPINS-*BLACT*$_{Mg}$ S728A
pOPINS-*BLACT*$_{Mg}$-S728A-Rv	GAGGAATTCACGCTCAAGACCATAACGCGCCC	pOPINS-*BLACT*$_{Mg}$ S728A
pOPINS-*OB*$_{Mg}$-Fw	GAACAGATCGGTGGTCATCAATCACGTCTCACGGGTGATCG	pOPINS-*OB*$_{Mg}$
pOPINS-*OB*$_{Mg}$-Rv	GTCTAGAAAGCTTTAGTGACCTGGTTCGGTACCCAATTTCAC	pOPINS-*OB*$_{Mg}$
pOPINS-*OB*$_{Mg}$-N103A-Fw	TATCCGGCGCGTGTGGTTTACACACTTGCCGCGCG	pOPINS-*OB*$_{Mg}$ N103A
pOPINS-*OB*$_{Mg}$-N103A-Rv	CACACGCGCCGGATACACACGAGGCTCTTCGATCAC	pOPINS-*OB*$_{Mg}$ N103A
pOPINS-OB$_{Mg}$-R112A-Fw	GCCGCGGCGGAAATTCGTCAAGGCGATTATCACAAGCG	pOPINS-*OB*$_{Mg}$ R112A
pOPINS-*OB*$_{Mg}$-R112A-Rv	AATTTCCGCCGCGGCAAGTGTGTAAACCACACGATTCGG	pOPINS-*OB*$_{Mg}$ R112A
pOPINS-*OB*$_{Mg}$-R115A-Fw	GAAATTGCGCAAGGCGATTATCACAAGCGTGTGCGCG	pOPINS-*OB*$_{Mg}$ R115A
pOPINS-*OB*$_{Mg}$-R115A-Rv	GCCTTGCGCAATTTCGCGCGCGGCAAGTGTGTAAA	pOPINS-*OB*$_{Mg}$ R115A
pOPINS-*OB*$_{Mg}$-H120A-Fw	GATTATGCGAAGCGTGTGCGCGAGAAAGTGCAAGTC	pOPINS-*OB*$_{Mg}$ H120A

pOPINS-*OB_{Mg}*-H120A-Rv	ACGCTTCGCATAATCGCCTTGAC GAATTTCGCGCG	pOPINS-*OB_{Mg}* H120A
pOPINS-*OB_{Mg}*-K121A-Fw	TATCACGCGCGTGTGCGCGAGA AAGTGCAAGTCGTAC	pOPINS-*OB_{Mg}* K121A
pOPINS-*OB_{Mg}*-K121A-Rv	CACACGCGCGTGATAATCGCCT TGACGAATTTCGCGC	pOPINS-*OB_{Mg}* K121A
pOPINS-*OB_{Mg}*-R122A-Fw	CACAAGGCGGTGCGCGAGAAAG TGCAAGTCGTACTGTATCGC	pOPINS-*OB_{Mg}* R122A
pOPINS-*OB_{Mg}*-R122A-Rv	GCGCACCGCCTTGTGATAATCG CCTTGACGAATTTCGCGC	pOPINS-*OB_{Mg}* R122A
pOPINS-*OB_{Mg}*-R144A-Fw	CTGTATGCGAGCGCGAAAGGTG GCGAGCCCGTGC	pOPINS-*OB_{Mg}* R144A
pOPINS-*OB_{Mg}*-R144A-Rv	CGCGCTCGCATACAGTACGACT TGCACTTTCTCGCGC	pOPINS-*OB_{Mg}* R144A
pOPINS-*OB_{Mg}*-R150A-Fw	GTACTGGCGGTGCATGGTCAAC TTGCCGCACCCC	pOPINS-*OB_{Mg}* R150A
pOPINS-*OB_{Mg}*-R150A-Rv	ATGCACCGCCAGTACATCACCA TAACGATAGAGCACGGG	pOPINS-*OB_{Mg}* R150A
pOPINS-*OB_{Mg}*-H152A-Fw	CGTGTGGCGGGTCAACTTGCCG CACCCCCAGCTG	pOPINS-*OB_{Mg}* H152A
pOPINS-*OB_{Mg}*-H152A-Rv	TTGACCCGCCACACGCAGTACA TCACCATAACGATAGAGCACGG	pOPINS-*OB_{Mg}* H152A
pOPINS-*OB_{Mg}*-R162A-Fw	GCTGCGGCGAACCCCGGGGAG CTGGATTATCGTGCG	pOPINS-*OB_{Mg}* H162A
pOPINS-*OB_{Mg}*-R162A-Rv	GGGGTTCGCCGCAGCTGGGGG TGCGGCAAGTTGAC	pOPINS-*OB_{Mg}* H162A
pOPINS-*OB_{Mg}*-R170A-Fw	GATTATGCGGCGTATCTTGCAC GTCAATACATTTACAATCGC	pOPINS-*OB_{Mg}* R170A
pOPINS-*OB_{Mg}*-R170A-Rv	ATACGCCGCATAATCCAGCTCC CCGGGGTTGCG	pOPINS-*OB_{Mg}* R170A
pOPINS-*OB_{Mg}*-R175A-Fw	CTTGCAGCGCAATACATTTACAA TCGCATGCTTATTGACAATCCC	pOPINS-*OB_{Mg}* R175A
pOPINS-*OB_{Mg}*-R175A-Rv	GTATTGCGCTGCAAGATACGCA CGATAATCCAGCTCCC	pOPINS-*OB_{Mg}* R175A
pOPINS-*OB_{Mg}*-R188A-Fw	AATCCCGCGGCTATCGTGAAATT GGGTACCGAACC	pOPINS-*OB_{Mg}* R188A
pOPINS-*OB_{Mg}*-R188A-Rv	GATAGCCGCGGGATTGTCAATA AGCATGCGATTGTAAATGTATTG ACG	pOPINS-*OB_{Mg}* R188A
pOPINS-*OB_{Mg}*-K192A-Fw	ATCGTGGCGTTGGGTACCGAAC CAGGTCACTAAAGCTTTC	pOPINS-*OB_{Mg}* R192A
pOPINS-*OB_{Mg}*-K192A-Rv	ACCCAACGCCACGATAGCACGG GGATTGTCAATAAGCATG	pOPINS-*OB_{Mg}* R192A
pOPINS-*OB_{Mg}*-R112A/R115A/R122A-Fw	GCCGCGGCGGAAATTGCGCAAG GCGATTATCACAAG	pOPINS-*OB_{Mg}* R112A/R115A/R122A
pOPINS-*OB_{Mg}*-R112A/R115A/R122A-Rv	AATTTCCGCCGCGGCAAGTGTG TAAACCACACGATTCGG	pOPINS-*OB_{Mg}* R112A/R115A/R122A
pOPINS-*OB_{Mg}*-R150A/R188A/K192A-Fw	AATCCCGCGGCTATCGTGGCGT TGGGTACCGAACC	pOPINS-*OB_{Mg}* R150A/R188A/K192A
pOPINS-*OB_{Mg}*-R150A/R188A/K192A-Rv	GATAGCCGCGGGATTGTCAATA AGCATGCGATTGTAAATGTATTG ACG	pOPINS-*OB_{Mg}* R150A/R188A/K192A
pOPINS-*OB_{Mg}*-linearise-Fw	TAAAGCTTTCTAGACCATTTAAA CACCACCACCACC	pOPINS-*OB_{Mg}* fragment
pOPINS-*OB_{Mg}*-linearise-Rv	GTGACCTGGTTCGGTACCCAAT TTCACG	pOPINS-*OB_{Mg}* fragment

pOPINS-*OB-BLACT*-Fw	ACCGAACCAGGTCACCAAGAAGAGCTGAAAGTCACTTTCATCGATG	pOPINS-*OB-BLACT*
pOPINS-*OB-BLACT*-Rv	GTCTAGAAAGCTTTAAATCGTTGTGTTGACTTGCCAACGC	pOPINS-*OB-BLACT*
pOPINS-*OB-BLACT*-linearise-Fw	GCGAACAGATCGGTGGTCAAGAAGAGCTGAAAGTCACTTTCATCGATGTTGG	pOPINS-*OB-BLACT* fragment
pOPINS-*OB-BLACT*-linearise-Rv	GTGACCTGGTTCGGTACCCAATTTCACG	pOPINS-*OB-BLACT* fragment
pOPINS-*OB-MBP-BLACT*-Fw	ACCGAACCAGGTCACGGCAGCAGCGGCAGCAGCAAAATCGAAGAAGGTAAACTGGTAATCTGG	pOPINS-*OB-MBP-BLACT*
pOPINS-*OB-MBP-BLACT*-Rv	TTTCAGCTCTTCTTGGCTGCTGCCGCTGCTGCCTTTCGTGATGCGAGTCTGCGCGTCTTTCAGGGC	pOPINS-*OB-MBP-BLACT*
pOPINS-*MBP-BLACT*-linearise-Fw	GCAAAATCGAAGAAGGTAAACTGGTAATCTGG	pOPINS-*MBP-BLACT* fragment
pOPINS-*MBP-BLACT*-linearise-Rv	ACCACCGATCTGTTCGCGATGCGCTTCAATAA	pOPINS-*MBP-BLACT* fragment
pOPINS-*ComEA-MBP-BLACT*-Fw	ATCGCGAACAGATCGGTGGTGCTAGAAAGATTAACATCAATACC	pOPINS-*ComEA-MBP-BLACT*
pOPINS-*ComEA-MBP-BLACT*-Rv	TTTACCTTCTTCGATTTTGCTGCTGCCGCTGCTGCCGCGAACAGTAATCAGATCTTTCAA	pOPINS-*ComEA-MBP-BLACT*
pOPINS-*Sac7d-MBP-BLACT*-Fw	ATCGCGAACAGATCGGTGGTATGGTGAAGGTGAAATTTAAATATAAGGGA	pOPINS-*Sac7d-MBP-BLACT*
pOPINS-*Sac7d-MBP-BLACT*-Rv	TTTACCTTCTTCGATTTTGCTGCTGCCGCTGCTGCCCTTTTTTTCACGTTCCGCGCGGGC	pOPINS-*Sac7d-MBP-BLACT*
Biophysical assays#		
5'OH-5'FAM*	T*GGGTAGGATCATCAGTAATAAGGATAGTGGGAAAGCTCACAGACCACCT	Nuclease assay
5'Phos-5'FAM*	PT*GGGTAGGATCATCAGTAATAAGGATAGTGGGAAAGCTCACAGACCACCT	Nuclease assay
5'OH-5'FAM*-(PTO)	T*/G/G/G/T/A/G/G/A/T/C/A/T/C/A/G/T/A/A/T/A/A/G/G/A/T/A/G/T/G/G/G/A/A/A/G/C/T/C/A/C/A/G/A/C/C/A/C/C/G	Nuclease assay
5'Phos-5'FAM*-(PTO)	PT*/G/G/G/T/A/G/G/A/T/C/A/T/C/A/G/T/A/A/T/A/A/G/G/A/T/A/G/T/G/G/G/A/A/A/G/C/T/C/A/C/A/G/A/C/C/A/C/C/G	Nuclease assay
5'OH-3'FAM£-(PTO)	C/G/G/G/T/A/G/G/A/T/C/A/T/C/A/G/T/A/A/T/A/A/G/G/A/T/A/G/T/G/G/G/A/A/A/G/C/T/C/A/C/A/G/A/C/C/A/C/C/T£	Nuclease assay
5'OH-5'FAM*-(PTO)	T*/G/G/G/T/A/G/G/A/T/C/A/T/C/A/G/T/A/A/T/A/A/G/G/A/T/A/G/T/G/G/G/A/A/A/G/C/T/C/A/C/A/G/A/C/C/A/C/C/G	Nuclease assay
5'FAM$	$GTTCGCAACGAA	Fluorescence anisotropy
5'FAM$-(PTO)	$G/T/T/C/G/C/A/A/C/G/A/A	Fluorescence anisotropy

[#] The complementary strand for dsDNA probes is not shown. Only one of the two strands is FAM-labelled. Unless stated otherwise, all DNA probes for nuclease assays are 50 nt in length, and all probes for fluorescence anisotropy are 12 nt in length.
* FAM was attached to the first base (thymine, T) at the 5′ terminus.
[£] FAM was attached to the last base (thymine, T) at the 3′ terminus.
[$] FAM was attached to the 5' phosphate group.
[p] 5' phosphorylation modification.
/ The uncleavable phosphorothioate bond.
/ The phosphodiester bond.

References

1. Pei,X.-Y., Bralley,P., Jones,G.H. and Luisi,B.F. (2015) Linkage of catalysis and 5′ end recognition in ribonuclease RNase J. *Nucleic Acids Res.*, **43**, 8066–8076.

2. Sierra-Gallay,I.L. de la, Mathy,N., Pellegrini,O. and Condon,C. (2006) Structure of the ubiquitous 3′ processing enzyme RNase Z bound to transfer RNA. *Nat. Struct. Mol. Biol.*, **13**, 376–377.

3. Hermoso,J.A., Lagartera,L., González,A., Stelter,M., García,P., Martínez-Ripoll,M., García,J.L. and Menéndez,M. (2005) Insights into pneumococcal pathogenesis from the crystal structure of the modular teichoic acid phosphorylcholine esterase Pce. *Nat. Struct. Mol. Biol.*, **12**, 533–538.

4. Sierra-Gallay,I.L. de la, Pellegrini,O. and Condon,C. (2005) Structural basis for substrate binding, cleavage and allostery in the tRNA maturase RNase Z. *Nature*, **433**, 657–661.

5. Tao,Y., Budhipramono,A., Huang,J., Fang,M., Xie,S., Kim,J., Khivansara,V., Dominski,Z., Tong,L., Brabander,J.K.D., *et al.* (2024) Anticancer benzoxaboroles block pre-mRNA processing by directly inhibiting CPSF3. *Cell Chem. Biol.*, **31**, 139-149.e14.

6. Abramson,J., Adler,J., Dunger,J., Evans,R., Green,T., Pritzel,A., Ronneberger,O., Willmore,L., Ballard,A.J., Bambrick,J., *et al.* (2024) Accurate structure prediction of biomolecular interactions with AlphaFold 3. *Nature*, **630**, 493–500.

7. Ashkenazy,H., Abadi,S., Martz,E., Chay,O., Mayrose,I., Pupko,T. and Ben-Tal,N. (2016) ConSurf 2016: an improved methodology to estimate and visualize evolutionary conservation in macromolecules. *Nucleic Acids Res.*, **44**, W344–W350.

8. Keene,O.N. (1995) The log transformation is special. *Stat. Med.*, **14**, 811–819.

9. Galleni,M., Lamotte-Brasseur,J., Rossolini,G.M., Spencer,J., Dideberg,O., Frère,J.-M. and Group,T.M.-β-L.W. (2001) Standard Numbering Scheme for Class B β-Lactamases. *Antimicrob. Agents Chemother.*, **45**, 660–663.

10. Paradzik,T., Ivic,N., Filic,Z., Manjasetty,B.A., Herron,P., Luic,M. and Vujaklija,D. (2013) Structure–function relationships of two paralogous single-stranded DNA-binding proteins from *Streptomyces coelicolor*: implication of SsbB in chromosome segregation during sporulation. *Nucleic Acids Res.*, **41**, 3659–3672.

11. Madru,C., Martínez-Carranza,M., Laurent,S., Alberti,A.C., Chevreuil,M., Raynal,B., Haouz,A., Meur,R.A.L., Delarue,M., Henneke,G., *et al.* (2023) DNA-binding mechanism and evolution of replication protein A. *Nat. Commun.*, **14**, 2326.

12. Huang,C.-Y., Hsu,C.-H., Sun,Y.-J., Wu,H.-N. and Hsiao,C.-D. (2006) Complexed crystal structure of replication restart primosome protein PriB reveals a novel single-stranded DNA-binding mode. *Nucleic Acids Res.*, **34**, 3878–3886.

13. Pan,J., Singh,A., Hanning,K., Hicks,J. and Williamson,A. (2024) A role for the ATP-dependent DNA ligase lig E of *Neisseria gonorrhoeae* in biofilm formation. *BMC Microbiol.*, **24**, 29.

14. Tumbale,P.P., Jurkiw,T.J., Schellenberg,M.J., Riccio,A.A., O'Brien,P.J. and Williams,R.S. (2019) Two-tiered enforcement of high-fidelity DNA ligation. *Nat. Commun.*, **10**, 5431.

15. Zuckman,D.M., Hung,J.B. and Roy,C.R. (1999) Pore-forming activity is not sufficient for *Legionella pneumophila* phagosome trafficking and intracellular growth. *Mol. Microbiol.*, **32**, 990–1001.

16. Kolter,R., Inuzuka,M. and Helinski,D.R. (1978) Trans-complementation-dependent replication of a low molecular weight origin fragment from plasmid R6K. *Cell*, **15**, 1199–1208.

17. Berger,K.H. and Isberg,R.R. (1993) Two distinct defects in intracellular growth complemented by a single genetic locus in *Legionella pneumophila*. *Mol. Microbiol.*, **7**, 7–19.

18. Braus,S.A.G., Short,F.L., Holz,S., Stedman,M.J.M., Gossert,A.D. and Hospenthal,M.K. (2022) The molecular basis of FimT-mediated DNA uptake during bacterial natural transformation. *Nat Commun*, **13**, 1065.

19. Chen,J., Felipe,K.S. de, Clarke,M., Lu,H., Anderson,O.R., Segal,G. and Shuman,H.A. (2004) *Legionella* Effectors That Promote Nonlytic Release from Protozoa. *Science*, **303**, 1358–1361.

20. Assenberg,R., Delmas,O., Graham,S.C., Verma,A., Berrow,N., Stuart,D.I., Owens,R.J., Bourhy,H. and Grimes,J.M. (2008) Expression, purification and crystallization of a lyssavirus matrix (M) protein. *Acta Crystallogr. Sect. F*, **64**, 258–262.
